# Nanofocusing of X-ray free-electron laser using wavefront-corrected multilayer focusing mirrors

**DOI:** 10.1038/s41598-018-35611-0

**Published:** 2018-11-28

**Authors:** S. Matsuyama, T. Inoue, J. Yamada, J. Kim, H. Yumoto, Y. Inubushi, T. Osaka, I. Inoue, T. Koyama, K. Tono, H. Ohashi, M. Yabashi, T. Ishikawa, K. Yamauchi

**Affiliations:** 1 Department of Precision Science and Technology, Graduate School of Engineering, Osaka University, 2-1 Yamada-oka, Suita, Osaka, 565-0871 Japan; 2 Pohang Accelerator Laboratory, Pohang, Gyeongbuk 37673 Republic of Korea; 3 Japan Synchrotron Radiation Research Institute, 1-1-1 Kouto, Sayo-cho, Sayo-gun, Hyogo 679-5198 Japan; 4 RIKEN SPring-8 Center, 1-1-1 Kouto, Sayo, Hyogo 679-5148 Japan; 5 Center for Ultra-Precision Science and Technology, Graduate School of Engineering, Osaka University, 2-1 Yamada-oka, Suita, Osaka, 565-0871 Japan

## Abstract

A method of fabricating multilayer focusing mirrors that can focus X-rays down to 10 nm or less was established in this study. The wavefront aberration induced by multilayer Kirkpatrick–Baez mirror optics was measured using a single grating interferometer at a photon energy of 9.1 keV at SPring-8 Angstrom Compact Free Electron Laser (SACLA), and the mirror shape was then directly corrected by employing a differential deposition method. The accuracies of these processes were carefully investigated, considering the accuracy required for diffraction-limited focusing. The wavefront produced by the corrected multilayer focusing mirrors was characterized again in the same manner, revealing that the root mean square of the wavefront aberration was improved from 2.7 (3.3) rad to 0.52 (0.82) rad in the vertical (horizontal) direction. A wave-optical simulator indicated that these wavefront-corrected multilayer focusing mirrors are capable of achieving sub-10-nm X-ray focusing.

## Introduction

Since the discovery of X-rays, X-ray sources have evolved from X-ray tubes to synchrotron radiation sources. X-ray free-electron lasers (XFELs) have now emerged due to the use of sophisticated accelerator techniques. FLASH^1^, SCSS^2^, and FERMI^3^ in the soft X-ray region and LCLS^4^, SACLA^5^, PAL-XFEL^6^, and the European XFEL^7^ in the hard X-ray region are already in operation. An XFEL itself can provide unprecedented peak brilliance roughly a billion times greater than that of a synchrotron X-ray source in the X-ray region. Furthermore, combining X-ray focusing optics can drastically enhance the performance. Thus, various facilities have competed in the development of high-performance X-ray focusing optics and the production of X-ray nanobeams with high peak power densities. Among the available focusing optics, such as diffractive and refractive lenses^8–16^ for X-rays, reflective lenses have the highest throughputs and resistances to radiation damage, as well as relatively long working distances^17^. Two types of total-reflection Kirkpatrick–Baez (KB) mirrors were developed to focus XFELs at SACLA in the early days of its operation: a simple 1 μm focusing system^18^ and a 50 nm focusing system^19^ that utilizes a two-stage focusing scheme for tightly focusing X-rays. The latter focusing system could reach a peak power density of 10^20^ W/cm^2^. These systems have been operated for users, resulting in many significant achievements. In particular, such ultradense electromagnet fields have enabled the observation of nonlinear phenomena in the hard X-ray region, such as double core-hole creation in Kr^20^, saturable absorption in Fe^21^, and two-photon absorption in Ge^22^. Furthermore, they enabled the realization of an atomic inner-shell laser in the hard X-ray region^23^.

However, focusing X-rays down to 10 nm or less using conventional total reflection mirrors is impossible, because mirrors with large numerical apertures (NAs) are necessary to focus beams so narrowly and large grazing incidence angles are required to increase the NAs of the mirrors. This approach is limited by the critical angle, which depends on the electron density of the surface material employed. In fact, the grazing incidence angles of focusing mirrors have already reached their limits. To overcome this restriction, the use of Bragg reflection based on multilayer structures is essential. Multilayer mirrors can have larger grazing incidence angles, depending on their multilayer period, than conventional total reflection mirrors. However, another problem occurs instead. The fabrication of such large-NA focusing mirrors is difficult because the allowable mirror shape fabrication errors decrease with increasing grazing incidence angle^24^. In particular, the metrology tools for profiling mirror shape errors suffer from not only the small mirror shape tolerance, but also difficulty in measuring the steeply curved shapes of large-NA focusing mirrors^25^. Common metrology tools for X-ray mirrors, such as optical interferometers and slope profilers, can barely determine steeply curved shapes with an accuracy of 1 nm.

One possible means of overcoming these difficulties is a scheme involving precise wavefront correction based on X-ray wavefront information. Mimura *et al*. achieved 7 nm X-ray focusing by utilizing wavefront measurements and employing a phase retrieval method and a fine deformable mirror as a phase compensator^24^. However, the deformable mirrors employed have unstable shapes, so some technical difficulties remain to be addressed before practical application will be feasible. Seiboth *et al*. proposed wavefront correction using a combination of a corrective phase plate and ptychography, resulting in the achievement of diffraction-limited focusing through the use of aberrated refractive lenses together with the corrective phase plate^26^. This strategy is very powerful and easy to use. However, the absorption of the phase plate reduces the throughput of the focusing optics. In addition, the narrow footprint of the phase plate due to the normal incidence limits the order of aberrations that can be corrected. Consequently, this method may not be compatible with X-ray mirrors that tend to have mid- and high-order aberrations induced during fabrication.

Another important point is how to determine wavefront aberrations. Wavefront sensing using ptychography^26,27^, grating interferometry^28,29^, speckle tracking technique^30^, phase retrieval method^24^, X-ray pencil beam method^31,32^, and Shack–Hartmann sensors^33^ has been reported on. Ptychography is one of the most promising methods of determining two-dimensional wavefronts. However, object positioning with an accuracy smaller than the beam size is required. Thus, this approach is not suitable for the combination of large-NA focusing optics and XFELs, where the XFEL spot position varies shot by shot. The phase retrieval and X-ray pencil beam methods also are incompatible with XFEL nanofocusing because they require precise beam profiling very near the focus, where the peak power density is sufficiently high to break an inserted object.

**Figure 1:**
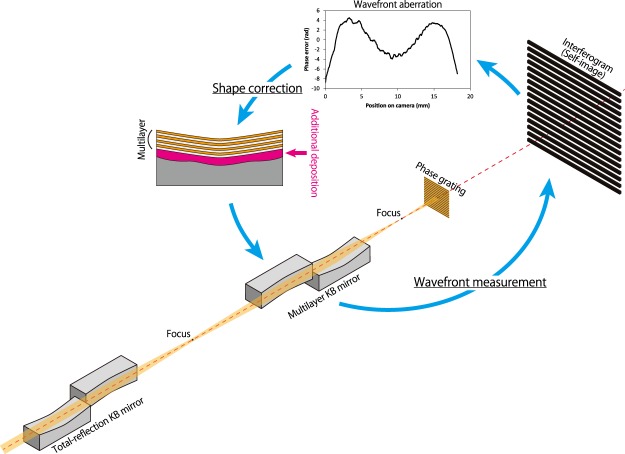
Schematic of the proposed scheme consisting of wavefront measurement and direct shape correction of the focusing mirrors.

In this study, we proposed and investigated a scheme based on wavefront determination using an X-ray single-grating interferometer and direct shape correction of focusing mirrors via a differential deposition method^34,35^ (see Fig. 1). Grating interferometers are very quick to use; a complete measurement can be performed via the Fourier transform method during a single exposure, and even the fringe scan method requires only a few image acquisitions. The simple setup, consisting of a grating and a camera, causes few systematic errors. This method is very insensitive to positioning error between the beam and the grating, meaning that it is robust against grating vibrations and not affected by the pointing stability of the beam. This robustness is the most important advantage of XFEL nanofocusing over the other methods. In addition, the direct mirror shape correction is advantageous because it does not entail photon loss, unlike the other phase compensators. The differential deposition method, which can be used for shape correction with atomic-level accuracy, is the most suitable for this purpose.

In this investigation, we demonstrated wavefront correction of multilayer focusing mirrors at SACLA to verify the effectiveness of the proposed scheme. The wavefront measurement and direct shape correction performances were carefully analysed, and the wavefront aberrations before and after correction were compared. In addition, the beam shape at the focus was characterized using a dark-field wire scanning method^36^, and the differences between the measured and reconstructed beam profiles were assessed.

## Two-stage Focusing Optics Based on Total-Reflection and Multilayer KB Mirrors

**Figure 2:**
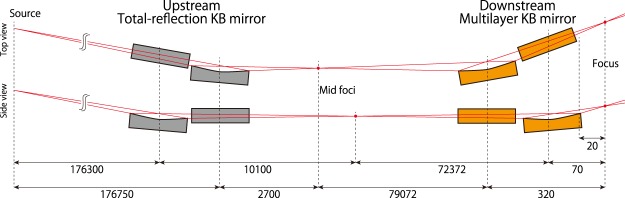
Geometrical arrangement of the two-stage focusing system. Length unit: mm.

Figure 2 shows the two-stage focusing optical system constructed and used in this study, which consists of an upstream total-reflection KB mirror system and a downstream multilayer KB mirror system. The detailed parameters of the mirrors are listed in Table 1. The upstream KB mirror weakly converges and diverges the XFEL beams, and the downstream KB mirror with a large acceptance tightly focuses the XFEL beams. This two-stage scheme has significant advantages that it can provide a very large NA and demagnification even when the working distance is relatively long, and can prevent radiation damage of the mirrors by receiving the intense X-rays in the large areas^19^. The upstream KB mirror has a grazing incidence angle approximately 10 times smaller than that of the downstream KB mirror. Therefore, the upstream KB mirror could be fabricated with a sufficient shape accuracy of 2 nm (whereas the required accuracy is 11 nm) via conventional fabrication processes, introducing negligibly small wavefront aberration. However, the downstream KB mirror requires a very high shape accuracy of less than 1 nm, making the fabrication of perfect mirrors by using the conventional techniques almost impossible. Thus, it can be anticipated that non-negligible wavefront aberration will be generated due to the downstream KB mirror.

**Table 1:** Parameters of the focusing mirrors.

Focusing direction	Upstream KB mirror		Downstream KB mirror	
	Vertical	Horizontal	Horizontal	Vertical
Coating	None	None	Multilayer	Multilayer
Average grazing incidence angle (mrad)	1.50	1.50	15.2	14.1
Effective mirror length (mm)	391.7	391.2	349.7	90.4
Focal length relative to mirror centre (m)	10.10	2.70	0.320	0.070
Individual demagnification (Total demagnification)	17.46	65.46	247.1 (16175.2)	1033.9 (18051.9)
Numerical aperture (×10^−3^)	0.029	0.109	9.96	11.88
Diffraction limit (nm, FWHM) @9.1 keV	2061	550	6.0	5.1

**Figure 3:**
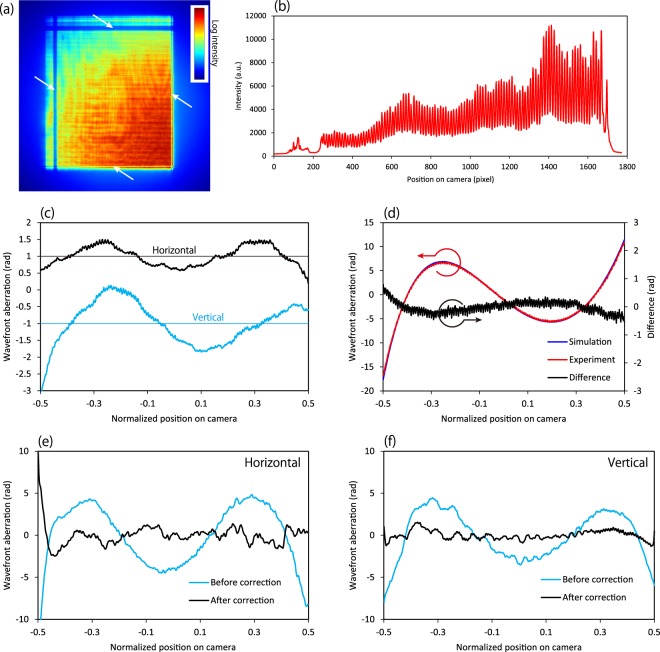
Wavefront measurements obtained using a single-grating interferometer. (**a**) Typical recorded self-image and (**b**) its cross-sectional profile. The white arrows in (**a**) indicated the uncoated area on the mirrors, used as position references in order to convert the wavefront data to shape error data. (**c**) Systematic errors induced by the camera. (**d**) Wavefront aberrations after introducing known comatic aberration by inclining the vertical focusing mirror by 10 μrad. The red and blue lines are the experimentally obtained results and those calculated using the Fresnel–Kirchhoff integral. The black line is the difference between the experimental and calculated results. (**e**,**f**) Wavefront aberrations obtained before and after direct shape correction. The term “normalized position on camera” in the graphs means the normalized position on the camera with respect to the effective bright field width, where the dimensions of the bright field at a camera length of 0.82 m were 16.4 × 19.4 mm^2^ (H × V).

Table 2 summarizes the design parameters of the Pt/C multilayer films employed, whose periods vary depending on the incident angle at each point on the mirror, i.e. they are laterally graded multilayers^37^. The multilayer films were produced using a specially developed magnetron sputtering deposition apparatus^37^, which can precisely control the workpiece scanning speed by utilizing a one-dimensional (1D) scanning stage and a computer and can deposit films with arbitrary thickness distributions based on control of the dwell time distribution. Reflectivity tests at BL29XUL EH4 (1 km downstream from the undulator) of SPring-8, where an incident X-ray beam with a photon energy of 9.1 keV was monochromatized with a Si 111 double-crystal monochromator, showed that the average reflectivity of the double reflection was 34.2%. This result is reasonable considering the interface roughness measured by a commercial X-ray reflectivity (XRR) instrument (Rigaku Corp., SmartLab 9KW) at the X-ray energy of the Cu Kα_1_ line. Each mirror had two uncoated stripes at the upstream and downstream ends to ensure perfect correspondence between the mirror and wavefront coordinates, as was essential for actual shape correction (see Fig. 3(a)).

**Table 2:** Design parameters of the multilayer films.

	Horizontal focusing	Vertical focusing
Targeted X-ray energy (keV)	9.1	9.1
Material	Pt/C	Pt/C
Number of periods	30	30
Maximum *d*-space(nm)	7.25	9.10
Minimum *d*-space (nm)	3.12	2.87
Thickness ratio (Gamma)	0.5	0.5

## Wavefront Measurement Using a Grating Interferometer

A typical self-image and its cross-sectional profile are shown in Fig. 3(a,b), respectively. Very fine and sufficiently high-contrast fringes are observable. A single-grating interferometer is expected to have few systematic errors due to its very simple configuration, consisting of only a grating and a camera. However, some systematic errors caused by slight distortions of the grating and camera may exist. Our previous studies^38^ revealed that sophisticated phase gratings introduce no systematic errors, meaning that they have uniform periodic patterns. However, the camera employed in this study introduced non-negligible systematic errors due to the intrinsic distortion of its components, such as the imaging device and/or lenses (Fig. 3(c)). A novel method of reconstructing consistent wavefronts using multiple wavefront data obtained by shifting the camera position could provide systematic-error-free wavefront aberration^38^. In addition, the accuracy of the interferometer was carefully tested by introducing known wavefront aberration into the focusing optical system. In general, an object with a known phase distribution, i.e. known thickness and density distributions may be inserted for this purpose. However, it is difficult to characterize these distributions perfectly in advance. In this study, comatic aberration that looks like the third-order polynomial was used, because its amount could be precisely controlled by adjusting the incidence angle error of the focusing mirror. In this case, the multilayer KB mirror was inclined by 10 μrad carefully by using an autocollimator. Figure 3(d) compares the comatic aberrations of the vertical focusing mirror that were measured and calculated using the Fresnel–Kirchhoff integral with the design parameters of the mirrors and interferometer. The two sets of results completely overlap and are consistent with each other, meaning that our interferometer can measure wavefront aberrations accurately. Finally, the actual wavefront aberrations of the multilayer mirrors, which were caused by deviations of the substrate shape from the designed ellipse, could be determined after subtracting the comatic aberration function and systematic errors induced by the camera (Fig. 3(e,f)) (see Methods for details).

## Aberration Correction

**Figure 4:**
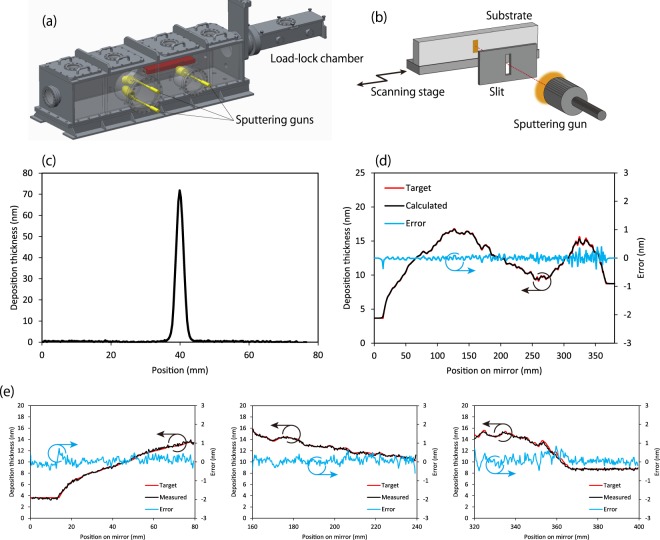
Differential deposition method. (**a**) Magnetron sputtering deposition apparatus, which was also employed for multilayer film deposition on the mirror. (**b**) Schematic of the inside of the chamber. A test substrate is exposed to sputtered Pt atoms through the slit with 2 mm clearance while scanning the substrate. (**c**) Measured stationary Pt deposition spot (exposure: 20 min). (**d**) Deposition thickness distribution for the horizontal focusing mirror. The red line is the target deposition thickness distribution calculated based on the wavefront aberration. The black line is the deposition thickness expected based on the actual dwell time distribution and the stationary deposition spot. (**e**) Comparison of the measured and target deposition thicknesses on test pieces. The target is the same as that used to obtain (**d**), and the horizontal coordinate corresponds to the horizontal coordinate in (**d**).

The shape errors of the multilayer mirrors were calculated based on the obtained wavefront aberrations. The old multilayers were completely removed from the mirror surfaces using an etchant, and the substrate shapes were precisely corrected by employing the same magnetron sputtering deposition apparatus that was used to fabricate the multilayer films (Fig. 4). The performance was tested in advance using other flat test pieces under the same conditions that were employed for shape correction of the actual mirrors. The shapes before and after correction were measured using a Fizeau interferometer (Zygo Corp., GPI-XR). The results revealed that the deposition thickness distribution on the test pieces closely agreed with the input data with an accuracy of 0.2 nm (root mean square (rms)) (Fig. 4(d,e)). After the test, the mirrors were corrected in the same manner and then were covered with the same multilayers again. The quality and *d*-space of the multilayer films were confirmed by measuring small test pieces, adhered near both ends of the mirror substrate and covered with the same multilayer film, using the laboratory-based XRR instrument. It was determined that *d*-spaces were consistent with the designed value with an accuracy of 0.05 nm.

Wavefront measurements of the corrected mirrors were performed in the same manner to confirm the wavefront correction. Figure 3(e,f) show the wavefront aberration before and after correction. The aberration of the shorter mirror (for vertical focusing) was drastically improved from 2.7 rad to 0.52 rad in rms height, and that of the longer mirror (for horizontal focusing) was improved from 3.3 rad to 0.82 rad in rms height except in the outermost region, although it exhibited a slight unexpected shape correction error.

**Figure 5:**
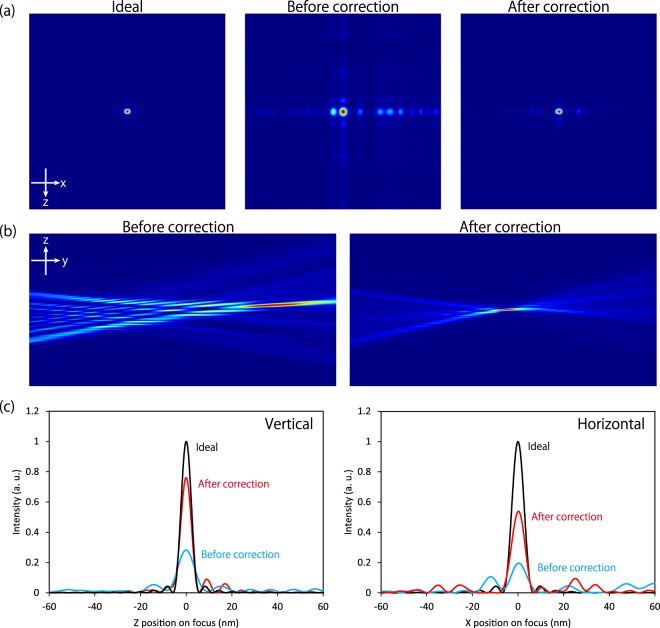
Focused beam intensity before and after correction, calculated using Fresnel–Kirchhoff integrals based on the corresponding wavefront aberrations. In the calculation, reflectivity distribution on the mirrors, calculated from the multilayer design and its performance evaluated in advance, was considered. (**a**) Beam intensity at the focus. Calculated area = 500 nm (x) × 500 nm (z). (**b**) Beam caustics in the vertical direction along the optical axis (y). Calculated area = 20 μm (y) × 500 nm (z). (**c**) Cross-section of the beam at the focus. The vertical axes were normalized by the maximum values of the ideal profiles for clear comparison of the relative peak heights.

Our wave-optical calculations based on Fresnel–Kirchhoff integrals were employed to assess whether the slight remaining shape errors affected the focusing profile. Figure 5(a,c) present the calculated beam intensity map and 1D profiles at the focus, respectively. Figure 5(b) shows the beam caustics along the optical axis before and after correction. The beam focusing state was drastically improved, and a sharply peaked beam with a full-width at the half maximum (FWHM) of 5.4 (6.7) nm in the vertical (horizontal) direction was obtained on the computer, meaning that the correction was successful. On the other hand, in the horizontal direction, some light converged not only at the main peak, but also in the side lobes, although the width of the main peak remained unchanged.

**Figure 6:**
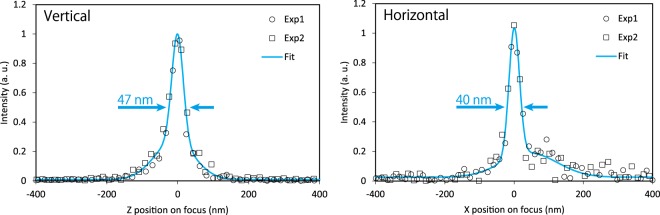
Measured beam intensity profiles at the focus. The profiling was performed twice (Exp1 and Exp2 in the graphs).

In the experiments, a dark-field wire scanning method was also employed for direct beam shape characterization (see Methods). Figure 6 shows the experimentally obtained beam profile at the focus. The FWHM was approximately 40–50 nm.

## Discussion

In this study, we established a precise mirror fabrication procedure by combining an accurate wavefront measurement method and a differential deposition method. High-performance multilayer focusing mirrors were successfully fabricated, which have the potential to achieve sub-10-nm X-ray focusing according to the measured wavefront quality. However, it was not possible to determine the very small beam size by performing direct beam profiling using a wire. We expect this failure to have resulted from the shot-by-shot source position fluctuation of 1 μm (σ); incident angle fluctuations of the multilayer and total reflection mirrors of a few tens of nanoradians (σ) and 50 nrad (σ), respectively, due to vibrations; translation vibrations of the wire and/or mirrors on the order of several nanometres (σ); and surface roughness of the wire, which was 5–10 nm (σ). The shot-by-shot source position fluctuation did not affect the beam position on the focal plane due to the very large demagnification. In addition, the fluctuation of the incident angles of the total reflection mirrors, which was observed 80 m downstream from the mirrors, could shift the virtual source position by 18 μm (σ), causing the focal point position to vary by 1 nm. On the other hand, the vibration of the incident angles of the multilayer mirrors may have influenced the beam position at the focus significantly, by 5–10 nm (σ), because the mirror focal length was relatively long, especially for the horizontal focusing mirror. In fact, the existence of relative vibrations between the wire and beam could be observed easily by monitoring the shot-by-shot fluctuated far-field images using the wire by placing an edge of the wire inside the focal spot. The combination of these factors seems to have broadened the apparent beam size.

This problem, i.e. poor pointing stability and/or vibration, is not critical for users performing general XFEL applications because uniform samples are employed or numerous samples are dispersed randomly. However, fine KB mirror adjustments, especially of the incident angles, perpendicularity of the mirrors, and astigmatism, require accurate beam shape characterization. To finish the adjustment perfectly in the above mentioned situations, single-shot beam shape characterization methods must be established, unlike the conventional methods that require object scanning and multiple XFEL shots. Possible candidates are saturable absorption measurement^21^, which can be used to estimate the degree of focus by detecting the nonlinear absorption coefficient, and speckle-size analysis^39^, which is based on the inversely proportional relationship between the irradiation area size and observed speckle size on a coherent diffraction pattern. We have already investigated the feasibility of these methods for tightly focused XFELs.

We anticipate that large-NA focusing mirrors, fabricated with the established techniques, will be available in the near future for users of XFEL and synchrotron radiation facilities, and that such tightly focused beams will open new frontiers in X-ray science.

## Methods

### XFEL experiments

The typical pulse energy at a photon energy of 9.1 keV at BL3 at SACLA was 300–400 μJ at the most upstream position. The repetition rate was 30 Hz. The band width and duration were estimated to be 30 eV and 8 fs, respectively^40^. Attenuators that could reduce the XFEL input by 2 × 10^−2^ times were used during wavefront measurement to avoid radiation damage of the grating. In addition, when the wire scan method was employed, the beams were typically attenuated by 5 × 10^−5^ times.

### Mirror substrate fabrication process

The substrate material was quartz glass. The substrates were roughly machined into elliptical shapes by conventional grinding and polishing. Then, they were finished by computer-controlled elastic emission machining (EEM)^41^. Our special optical interferometers, stitching interferometers^42,43^, were used for shape profiling. Mid- and high-spatial-frequency shape errors were effectively eliminated by several repetitions of shape correction using the EEM and interferometers.

### Laterally graded multilayer deposition

The laterally graded multilayer films were formed on the large-NA focusing mirrors using a specially developed DC magnetron sputtering deposition apparatus, which mainly consisted of two types of sputtering guns (Pt and C), a 1D scanning stage, a slit, and a load locking system (see Fig. 4(a))^37^. The sputtering targets with diameters of 2 in were placed 124 mm away from the workpiece. The sputtering conditions were as follows: Ar was used as a plasma gas; the input power was 20 W and 120 W for Pt and C, respectively; the gas pressure was 0.08 Pa; and the gas flow rate was 16 sccm and 10 sccm for Pt and C, respectively. The slit with 30 mm clearance was used to define the deposition area. The mirror substrate was subjected to sputtered Pt and C atoms sequentially while scanning the substrate based on the dwell time distribution calculated with the deconvolution algorithm.

### Single-grating interferometer

A λ/4 phase grating with period of 2.5 μm (NTT Advanced Technology Corporation) was installed 11.63 mm downstream from the focus. Self-images were recorded using an X-ray indirect camera (combination of ORCA-Flash4.0 and AA60, Hamamatsu Photonics) placed 820 mm downstream from the focus. It consists of a 10-μm-thick Gd_2_O_2_S:Tb scintillator, a mirror for visible light, two lenses, and a complementary metal oxide semiconductor (CMOS) camera. The camera had an effective field of view and pixel size of 27 × 27 mm^2^ and 13.2 μm/pixel, respectively. The four-step fringe scanning method was employed to analyse the interferograms. Each image was taken by accumulating 30 XFEL shots. The wavefront shapes were reconstructed using the general formulas^44^. The major quadratic function, representing the carrier fringe of the grating interferometer, was subtracted from the wavefront shape. Then, the remaining comatic aberration function was subtracted by fitting the function using the least-squares approach. The shape of the comatic aberration function was experimentally determined using multiple wavefront data obtained with different incident angles on the multilayer focusing mirror. The remaining component was the wavefront aberration caused by the shape error of the multilayer focusing mirrors. The repeatability of the wavefront measurements at SACLA was 0.2 rad (σ), which is sufficient to achieve the required wavefront sensing accuracy. The obtained wavefront aberrations were converted to shape errors on the mirrors, in which a simple ray-tracing calculation was used to determine a correspondence relationship between the coordinates on the mirror and on the wavefront data based on the uncoated areas on the mirror and on the wavefront data.

### Correction of systematic error of the grating interferometer caused by a camera

As explained in ref.^37^ 23 wavefront data points were acquired while scanning the camera in 1D with a scanning step of 118 (136) pixels in the horizontal (vertical) direction. The obtained data were aligned to centre the self-image. Corrected wavefront data were obtained by averaging the 23 data points. Figure 3(c) depicts the typical extracted systematic errors of the camera in the vertical and horizontal directions. It is assumed that the systematic errors were caused by manufacturing errors of the components of the used camera such as the lenses, mirrors, and CMOS camera and by misalignments of the components. However, the details have not been revealed yet.

### Differential deposition method

The differential deposition method is highly advantageous because a very stable deposition rate allows for shape correction with atomic-level accuracy. The same apparatus and conditions that were employed for multilayer formation were used. A Pt target was selected because the deposition rate of Pt is greater than that of C. A slit with a clearance of 2 mm was used to correct shape errors with spatial frequencies greater than 2 mm (Fig. 4(b,c)). The dwell time distribution used to generate the corresponding deposition thickness distribution was calculated with deconvolution of the target thickness distribution and stationary Pt deposition spot (Fig. 4(c,d)). To match the coordinates of the deposition system and the mirror, the position of the small stationary deposition spot on the mirror coordinate was measured. Based on the decided coordinates and the uncoated area position, our deposition system precisely corrected the mirror shape. Figure 4(d) reveals the close agreement between the target and simulated deposition thickness distributions, meaning that the deconvolution calculations were performed correctly. The typical performance of the method is shown in Fig. 4(e). It can provide at least 0.2 nm accuracy (rms). The observed high-spatial-frequency errors were caused by measurement errors. Thus, the actual errors would be smaller.

### Dark-field wire scanning method

The beam shape at the focus was characterized using the dark-field wire scanning method^36^. A cross Au wire with a diameter of 50 μm was placed at the focus. The bright-field X-rays were blocked by a beam stop located downstream from the focus. Only scattering X-rays from the wire surface were detected by a photodiode placed downstream from the beam stop. The X-ray signals were recorded while scanning the wire across the beam. The obtained curve represented the 1D beam shape profile because the scattering signal is proportional to the photon density of the beam.

## Data Availability

The datasets generated and/or analysed during this study are available from the corresponding author upon reasonable request.
